# High Counts of CD68+ and CD163+ Macrophages in Mantle Cell Lymphoma Are Associated With Inferior Prognosis

**DOI:** 10.3389/fonc.2021.701492

**Published:** 2021-08-30

**Authors:** Philippa Li, Ji Yuan, Fahad Shabbir Ahmed, Austin McHenry, Kai Fu, Guohua Yu, Hongxia Cheng, Mina L. Xu, David L. Rimm, Zenggang Pan

**Affiliations:** ^1^Department of Pathology, Yale University School of Medicine, New Haven, CT, United States; ^2^Department of Laboratory Medicine and Pathology, Mayo Clinic, Rochester, MN, United States; ^3^Department of Pathology and Microbiology, University of Nebraska Medical Center, Omaha, NE, United States; ^4^Department of Pathology, Wayne State University, Detroit, MI, United States; ^5^Department of Pathology and Laboratory Medicine, Roswell Park Cancer Center, Buffalo, NY, United States; ^6^Department of Pathology, Yantai Yuhuangding Hospital, Yantai, China; ^7^Department of Pathology, Shandong Provincial Hospital Affiliated to Shandong First Medical University, Jinan, China

**Keywords:** mantle cell lymphoma, lymphoma microenvironment, lymphoma associated macrophage, PD-L1, quantitative immunofluorescence analysis

## Abstract

**Background:**

Lymphoma-associated macrophages (LAMs) are key components in the lymphoma microenvironment, which may impact disease progression and response to therapy. There are two major subtypes of LAMs, CD68+ M1 and CD163+ M2. M2 LAMs can be transformed from M1 LAMs, particularly in certain diffuse large B-cell lymphomas (DLBCL). While mantle cell lymphoma (MCL) is well-known to contain frequent epithelioid macrophages, LAM characterization within MCL has not been fully described. Herein we evaluate the immunophenotypic subclassification, the expression of immune checkpoint molecule PD-L1, and the prognostic impact of LAMs in MCL.

**Materials and Methods:**

A total of 82 MCL cases were collected and a tissue microarray block was constructed. Immunohistochemical staining was performed using CD68 and CD163, and the positive cells were recorded manually in four representative 400× fields for each case. Multiplexed quantitative immunofluorescence assays were carried out to determine PD-L1 expression on CD68+ M1 LAMs and CD163+ M2 LAMs. In addition, we assessed Ki67 proliferation rate of MCL by an automated method using the QuPath digital imaging analysis. The cut-off points of optimal separation of overall survival (OS) were analyzed using the X-Tile software, the SPSS version 26 was used to construct survival curves, and the log-rank test was performed to calculate the *p*-values.

**Results:**

MCL had a much higher count of M1 LAMs than M2 LAMs with a CD68:CD163 ratio of 3:1. Both M1 and M2 LAMs were increased in MCL cases with high Ki67 proliferation rates (>30%), in contrast to those with low Ki67 (<30%). Increased number of M1 or M2 LAMs in MCL was associated with an inferior OS. Moreover, high expression of PD-L1 on M1 LAMs had a slightly better OS than the cases with low PD-L1 expression, whereas low expression of PD-L1 on M2 LAMs had a slightly improved OS, although both were not statistically significant.

**Conclusions:**

In contrast to DLBCL, MCL had a significantly lower rate of M1 to M2 polarization, and the high levels of M1 and M2 LAMs were associated with poor OS. Furthermore, differential PD-L1 expressions on LAMs may partially explain the different functions of tumor-suppressing or tumor-promoting of M1 and M2 LAMs, respectively.

## Introduction

Mantle cell lymphoma (MCL) accounts for 5-6% of non-Hodgkin lymphomas, with characteristic expression of cyclin D1 due to *CCND1-IGH* gene rearrangement. MCL has a broad morphologic spectrum with variable architectural patterns and cytologic features, which are associated with heterogeneous clinical behaviors (1–3). Patients with MCL usually present at advanced stages with an aggressive clinical course. The long-term prognosis remains poor with a median overall survival of 3-5 years despite significant improvement in the management (4). On the other hand, ~15% cases of MCL demonstrate indolent clinical course with an overall survival of 7-10 years (5–7). Therefore, it is necessary to identify the different prognostic subgroups of MCL and allow for risk-adjusted therapeutic approaches.

In recent years, studies focusing on tumor microenvironment and associated immunotherapies have been increasingly pursued, particularly in solid tumors including breast cancer, lung cancer and melanoma. Some of the most widely studied biomarkers for immunotherapy are the programmed death-1 receptor (PD-1, CD279) and its ligands PD-L1 (CD274, B7-H1) and PD-L2 (CD273, PDCD1LG2, B7-DC), which are essential in many autoimmune and neoplastic conditions (8–11). Interactions between PD-1 and PD-L1/PD-L2 induce immune evasion of tumor cells, which can be reversed by restoring effector T-cell functions through targeted therapy against PD-1 or its ligands (12, 13). In the hematopoietic system, PD-L1 is expressed in antigen-presenting cells and activated T-cells (12). Certain types of B-cell lymphomas may express PD-L1, including classic Hodgkin lymphoma (CHL), nodular lymphocyte-predominant Hodgkin lymphoma (NLPHL), and some diffuse large B-cell lymphoma (DLBCL) subtypes (14). Immunotherapies with PD-1 or PD-L1 blockade have shown clinical responses in these lymphomas (15).

Relatively few studies have assessed MCL immune microenvironment. In MCL, the lymphoma cells and microenvironment are thought to have low expression of PD-L1 (14, 16); however, it is not certain whether PD-L1 expression has any significance in clinical therapy and survival. In addition, immunotherapy in MCL has not provided desirable results. PD-L1 expression on MCL cells may induce suppression of anti-tumor immune responses. Therefore targeting PD-L1 on tumor cells may represent a novel approach to improve the efficacy of immunotherapy (17). Consequently, immunotherapy may be feasible in treating MCL and preventing lymphoma relapse.

Macrophages represent an essential component of tumor microenvironment, and a variable number of macrophages have been found in association with nearly all lymphoma types, which are referred as lymphoma-associated macrophages (LAMs) (18, 19). MCL is well-known for the presence of epithelioid histiocytes without phagocytic activities, so called “pink histiocytes” by many pathologists. LAMs have been divided into two major subtypes based on their immunophenotype, M1 and M2. Their functions are thought to be variable among different lymphoma types. M1 LAMs are considered to prevent the growth of tumors, whereas M2 LAMs are associated with angiogenesis and tumor progression (20). The presence of a high number of LAMs has been associated with aggressive clinical course in CHL, DLBCL, follicular lymphoma (FL) and angioimmunoblastic T-cell lymphoma (AITL) (18, 19, 21, 22). However, the significance of LAMs in MCL has not been fully characterized (18). Only a few studies have linked macrophage number with the prognosis of MCL, and the data on functional roles for LAMs in MCL are limited. Therefore, further studies are necessary to explore the characteristics and biological functions of LAMs in MCL. In this study, we investigated the number, subtype, and PD-L1 expression of LAMs in MCL and assessed their prognostic impact.

## Materials and Methods

### Case Selection and Data Collection

The pathology archives from two institutions (Yale University and University of Nebraska Medical Center) were retrospectively searched to identify cases of MCL from 2000 to 2019 after approval from local institutional review board (IRB# 2000023891). No individual patient consent was required. Diagnosis of MCL was based on the following major criteria: 1) Morphology: small cell, classic, and blastoid variants (blastic and pleomorphic); diffuse and nodular growth patterns; 2) Immunophenotype, particularly expression of CD5, cyclin D1, and SOX11; and 3) Molecular genetic studies for *CCND1* rearrangement if necessary. The major inclusion criteria of case selection included: 1) All *de novo* cases without prior treatment; 2) Locations: lymph node, gastrointestinal tract, spleen, and other solid organs; 3) Sufficient clinical data availability, including clinical information at diagnosis, treatment plans, follow-up, and survival data; and 4) Excisional or large biopsies. The exclusion criteria included: 1) *In-situ* mantle cell neoplasm, and MCL with mantle zone growth pattern; 2) Core biopsy, bone marrow biopsy, and decalcified specimens; 3) Suboptimal specimens with inadequate fixation, poor processing, or marked crush artifacts; 4) Inadequate remaining tissue in paraffin blocks; and 5) Insufficient clinical or pathology data.

The essential clinical information of each patient was collected, including age, gender, biopsy site(s), extent of disease by imaging studies, bone marrow biopsy results, clinical stage and status (ECOG and sMIPI), treatment regimens, responses, and outcomes. For all eligible cases, the pathology reports, H&E slides, and immunohistochemical slides were reviewed to confirm the diagnosis, in conjunction with flow cytometric results, molecular assays, and cytogenetic studies. The detailed clinical and pathologic characteristics are summarized in **Supplementary Tables 1, 2**.

### Construction of Tissue Microarray

A total of 82 eligible MCL cases were included in the study, and the formalin-fixed paraffin embedded (FFPE) tissue from each case was collected for tissue microarray (TMA). The H&E slides were reviewed to select the paraffin blocks with adequate tumor tissue for TMA construction. For each case, the lymphoma tissue was punched in duplicate (1.0 mm in diameter) and separately plated into one TMA block.

### Immunohistochemical Stains and Manual Evaluation

The TMA block was sectioned at 4.0 um thick. Immunohistochemical staining was performed on the sections according to the manufacture’s manual. The antibodies used in this study included CD68 (Clone PG-M1; DAKO, Carpinteria, CA, USA), CD163 (Clone 10D6; Abcam, Cambridge, MA, USA), cyclin D1 (Clone SP4; Cell Marque, Rocklin, California), and SOX11 (Clone MRQ-58; Cell Marque). The immunostains were performed on a Ventana Benchmark Ultra immunostainer (Ventana Medical Systems, Tucson, AZ, USA), with appropriate positive and negative controls. Immunohistochemical stains for CD68 and CD163 were evaluated in a quantitative method by recording positive cells manually in four representative 400× fields on the two 1.0 mm cores of each case.

### QuPath Digital Image Analysis

The immunostained slide for Ki67 (Clone MIB1; DAKO) was scanned using the Aperio ScanScope CS2 platform (Leica Biosystems, Inc., Buffalo Grove, IL, USA). The slide was scanned at 200× with a pixel size of 0.4986 µm × 0.4986 µm, which was analyzed using the QuPath software (https://qupath.github.io) to quantitatively calculate the Ki67 proliferative rate of the positive cells over all nucleated cells.

### Multiplexed Quantitative Immunofluorescence Assays

The TMA sections were briefly deparaffinized, followed by antigen retrieval at 97°C with pH 8.0 EDTA buffer for 20 minutes using PT module epitope retrieval solutions (Lab Vision, Waltham, MA, USA). Subsequently, a 30-minute incubation in 2.5% hydrogen peroxide was performed to block endogenous peroxidases and then unspecific antigens were blocked using a 0.3% BSA for 30 minutes. A multiplexed immunofluorescence staining was performed with three primary antibodies, including CD68 (Clone PG-M1; 1:200; DAKO), CD163 (Clone 10D6; 1:7500; Abcam), and PD-L1 (Clone SP142; 1:800; Abcam) on the same tissue section. Horseradish peroxidases (HRP)-conjugated secondary antibodies specific to each primary antibody isotype were used sequentially (anti-mouse IgG1, 1:100, eBioscience; anti-rabbit EnVision, DAKO; anti-mouse IgG3, 1:1,000, Abcam). Tyramide-bound fluorophores were added after each secondary antibody to bind to the HRPs. Specifically, Cyanine 7 tyramide (Cy7), cyanine 3 tyramide (CY3) tyramide, and cyanine 5 tyramide (Cy5) were used for CD68, CD163 and PD-L1, respectively. Finally, the nuclei were stained with 4,6-diamidino-2-phenylin-dole (DAPI). Quantitative immunofluorescence assays were performed on the Vectra Polaris (Perkin Elmer, Inc., Waltham, MA, USA) automated fluorescence microscopy platforms. The resultant images were analyzed and quantified using the InForm Software (Perkin Elmer) on all tumor spots as described previously (23).

### Statistical Analysis

The cut-off points of optimal separation of overall survival were analyzed using the X-Tile software (24), the SPSS version 26 (IBM, Armonk, New York, USA) was used to construct survival curves, and the log-rank test was performed to calculate the *p*-values.

## Results

### Clinical and Pathology Characteristics

The clinical and pathology data of the 82 patients with MCL are briefly summarized in **Table 1**, and the detailed clinicopathologic characteristics are depicted in **Supplementary Tables 1, 2**. There was a male predominance with a male to female ratio of ~3:1, and the median age at diagnosis was 67 years (range, 37 to 92 years). Thirty percent (22/74) of patients had documented systemic symptoms, including fatigue, fever, and weight loss. Bone marrow involvement was detected in 81% (48/59) of patients. The majority (67/70; 96%) of patients presented at high clinical stages (III/IV). Fifty cases had sufficient data to calculate the sMIPI score, of which 18 (36%) cases were classified as low risk, 22 (44%) intermediate risk, and 10 (20%) high risk.

**Table 1:** Summary of the clinicopathologic data of the 82 patients with MCL.

Total case number****	82****
Median Age (Years)	67 (37-92)
Male/Female	61/21
B-Symptoms	22/74 (30%)
BM Involvement	48/59 (81%)
Advanced Clinical Stages (III/IV)	67/70 (96%)
sMIPI Risk	
Low	18/50 (36%)
Intermediate	22/50 (44%)
High	10/50 (20%)
Immunohistochemical Stains	
CD20	53/53 (100%)
CD5	45/50 (90%)
CD23	0/14 (0%)
Cyclin D1	79/80 (99%)
SOX11	69/79 (87%)
CD10	2/29 (7%)
BCL6	0/21 (0%)
* CCND1* FISH	28/32 (88%)
Treatment	
Chemo- and/or Radio-Therapy	67/75 (87%)
Watchful Waiting	8/75 (13%)
Reponses to Treatment	
Complete Remission	38/63 (60%)
Partial Response	13/63 (21%)
Persistent or Progressive	12/63 (19%)
Stem Cell Transplant	24/68 (35%)
Follow Up (Median, Months)	36 (1-221)
Outcome (Deceased)	46/81 (57%)

Of the 75 patients with clinical treatment information, 67 (87%) received chemotherapy and/or radiotherapy, and the remaining eight (13%) were under observation with no therapy. Sixty-three patients had treatment responses available; 38 patients (60%) achieved a complete remission, 13 (21%) yielded a partial response, and 12 (19%) had persistent or progressive disease. Following chemotherapy, 24/68 (35%) of patients received allogeneic hematopoietic stem cell transplant. Eight-one patients were followed up clinically with a median duration of 36 months (range 1-221 months), and 46 (57%) had died.

**Figure 1:**
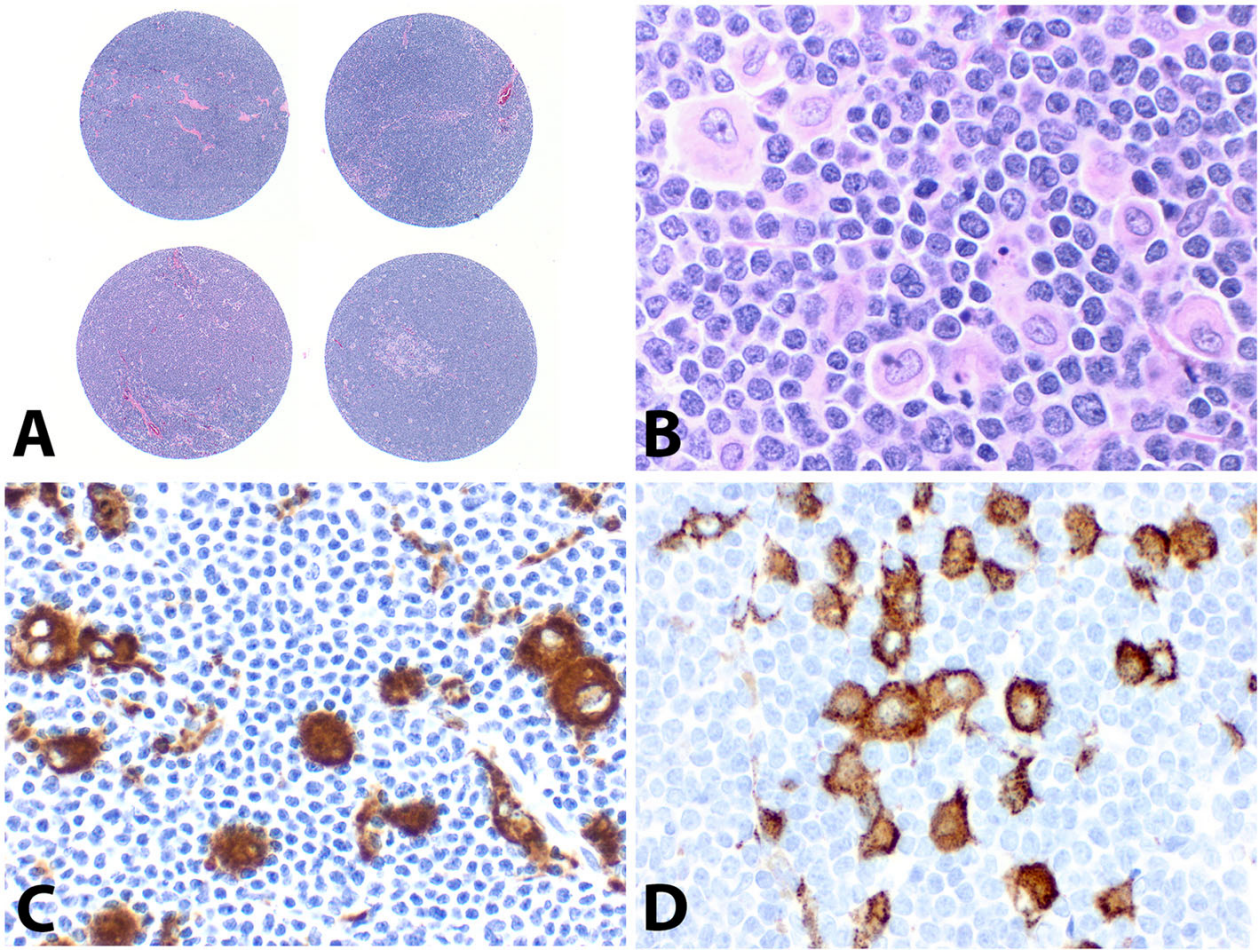
Construction of tissue microarray (TMA) and immunostains of CD68 and CD163. **(A)** Each MCL case had two separate 1.0 mm cores on the TMA (H&E, original magnification ×20). **(B)** Scattered epithelioid histiocytes admixed with abundant lymphoma cells (H&E, ×400), which were highlighted by immunostains with CD68 (**C**, ×400) and CD163 (**D**, ×400).

On the TMA block, each MCL case had two separate 1.0 mm cores with representative lymphoma tissue (**Figure 1A**). A variable number of epithelioid histiocytes were present admixed with tumor cells (**Figure 1B**). The vast majority of the MCL cases were positive for CD5 (45/50, 90%), cyclin D1 (79/80, 99%), and SOX11 (69/79, 87%); one case (case #60) was negative for both cyclin D1 and SOX11 without *CCND1* FISH data, which was excluded from further assays. Only two of 29 cases (7%) expressed CD10. BCL6 and CD23 were negative in 21 and 14 cases tested, respectively. FISH studies for *CCND1* rearrangement were performed in 32 cases, of which 28 (88%) cases were positive.

### Manual Counts of CD68+ and CD163+ Macrophages and Overall Survival

**Figure 2:**
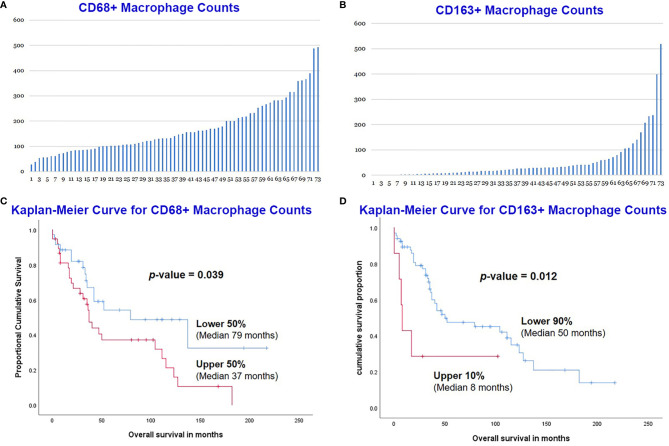
Manual count of CD68+ macrophages **(A)** and CD163+ macrophages **(B)** in the 73 MCL cases. MCL with lower CD68+ counts had a better overall survival (OS) than those with higher counts **(C)**. The lower count of CD163+ macrophage also predicted an improved OS **(D)**.

Immunohistochemical stains for CD68 and CD163 were performed on the TMA sections (**Figures 1C, D**), and the number of positive cells were recorded manually by a pathologist on four representative 400× fields for each case. A total of 73 MCLs were included in this assay after excluding nine cases with suboptimal staining or insufficient tissue. The average count for CD68+ cells was 170 (range 29-493) and CD163+ cells was 57 (range 0-519) (**Figures 2A, B**). The CD68+ macrophages were present in higher numbers than the CD163+ macrophages with an overall CD68:CD163 ratio of 3:1. In addition, CD163+ macrophages had a broader range and more frequent low counts.

The overall survival (OS) of the 73 MCL patients was calculated based on the CD68+ or CD163+ macrophages using the Kaplan-Meier analysis. The CD68+ macrophage cut-off was set to the median (50%) by X-Tile. The 50% of cases with lower CD68+ counts (n=36) had a significantly better OS than those 50% with higher counts (n=37) (**Figure 2C**). The CD163+ macrophage optimal cut-off was set to 90% by X-Tile, and the 90% of cases with lower (n=66) CD163+ macrophages had a better OS than the higher 10% (n=7) (**Figure 2D**).

### CD68, CD163, and PD-L1 Expression With Multiplexed Quantitative Immunofluorescence Analysis and Overall Survival

**Figure 3:**
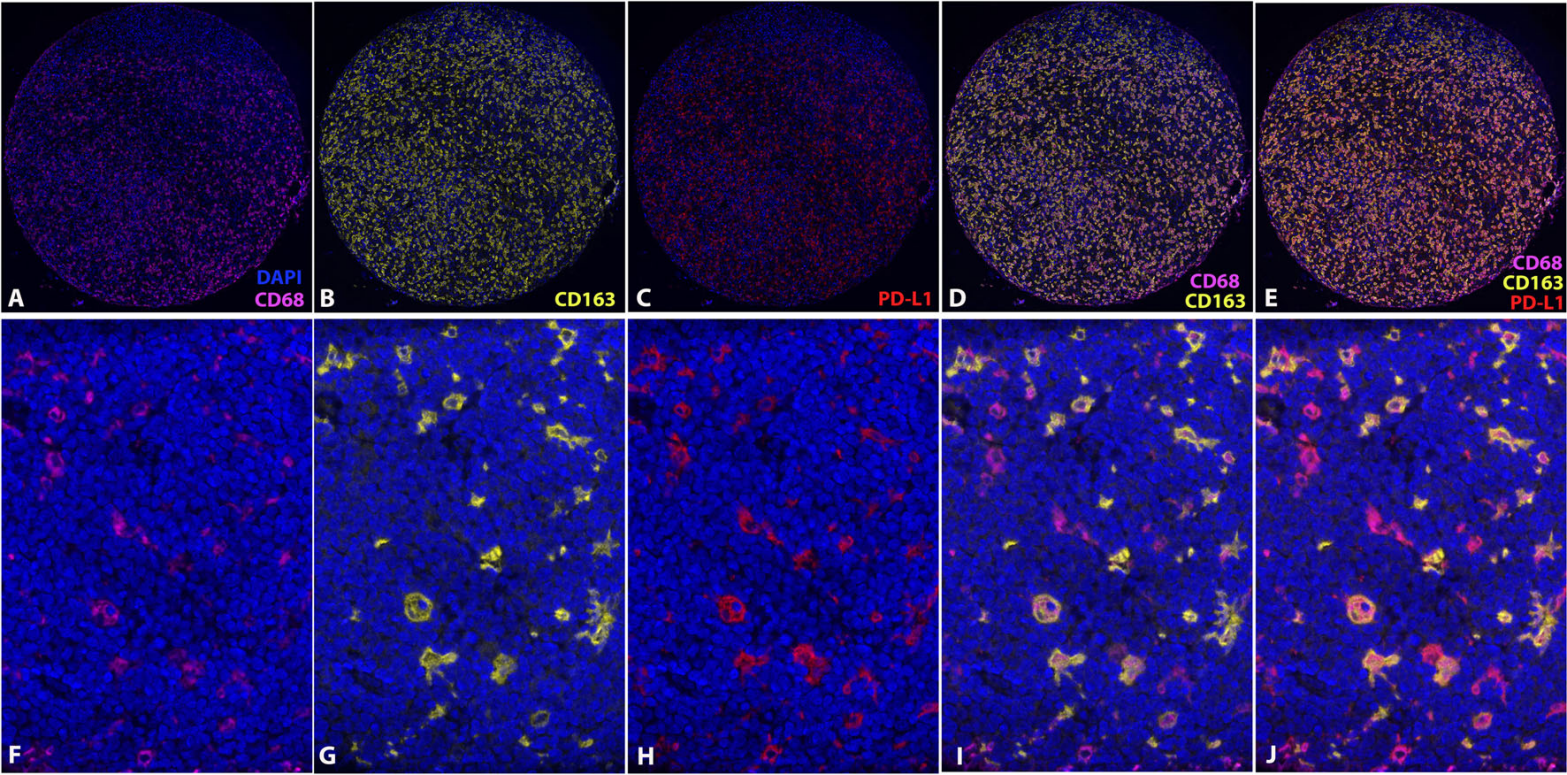
Multiplexed quantitative immunofluorescence analysis. Immunofluorescence stains of CD68 **(A, F)**, CD163 **(B, G)**, and PD-L1 **(C, H)**. Overlying of CD68 and CD163 **(D, I)**. Overlying of CD68, CD163 and PD-L1 **(E, J)**. (DAPI, counterstaining of nuclei; CD68, Cy7; CD163, Cy3; PD-L1, Cy5).

Expression of CD68, CD163, and PD-L1 was assessed on the TMA section with multiplexed quantitative immunofluorescence analysis, and the PD-L1 expression was co-localized with either CD68 or CD163 (**Figures 3A–J**). A total of 73 MCLs were included in this assay after excluding nine cases with poor staining or insufficient tissue.

**Figure 4:**
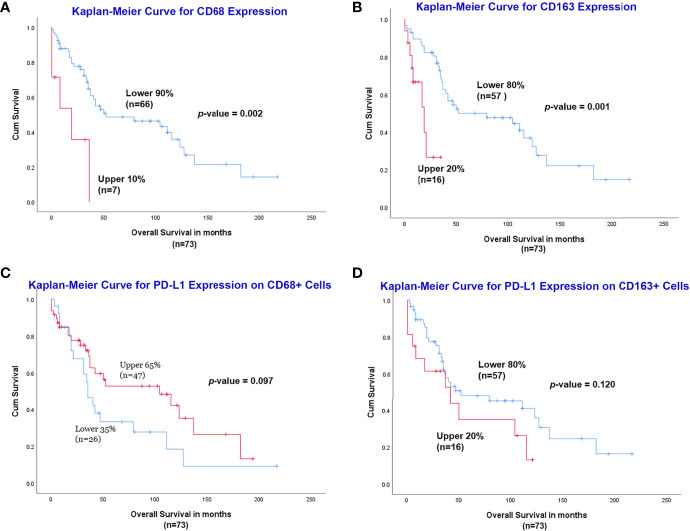
Assessment of CD68, CD163 and PD-L1 expression with multiplexed quantitative immunofluorescence analysis. Lower expression of CD68 **(A)** and CD163 **(B)** was associated with a significantly better outcome. Increased expression of PD-L1 on CD68+ cells had a slightly better OS **(C)**, while the high expression of PD-L1 on CD163+ cells was related to inferior prognosis **(D)**, although both were not statistically significant.

The optimal cut-off for survival curve of CD68 expression was set to 90% by X-Tile, and the 90% of cases with lower expression of CD68 (n=66) had a significantly better OS than the higher 10% (n=7) (*p*=0.002) (**Figure 4A**). Similarly, the optimal cut-off of CD163 expression was set to 80% by X-Tile, and the 80% of cases with lower expression of CD163 (n=57) had a significantly better OS than the higher 20% (n=16) (*p*=0.001) (**Figure 4B**).

For PD-L1 expression on CD68+ macrophages, the optimal cut-off for survival curve was set to 65% by X-Tile, and the 65% of cases (n=47) with higher expression of PD-L1 on CD68+ cells had a slightly better OS than the lower 35% (n=26) but it did not reach statistical significance (*p*=0.097) (**Figure 4C**). Expression of PD-L1 on CD163+ macrophages was measured and the optimal cut-off for survival curve was set to 80% by X-Tile; the 80% of cases (n=57) with lower expression of PD-L1 on CD163+ cells had a slightly better OS than the higher 20% (n=16) but it was not statistically significant (*p*=0.120) (**Figure 4D**).

### Assessment of Ki67 Proliferation Index With Digital Image Analysis

**Figure 5:**
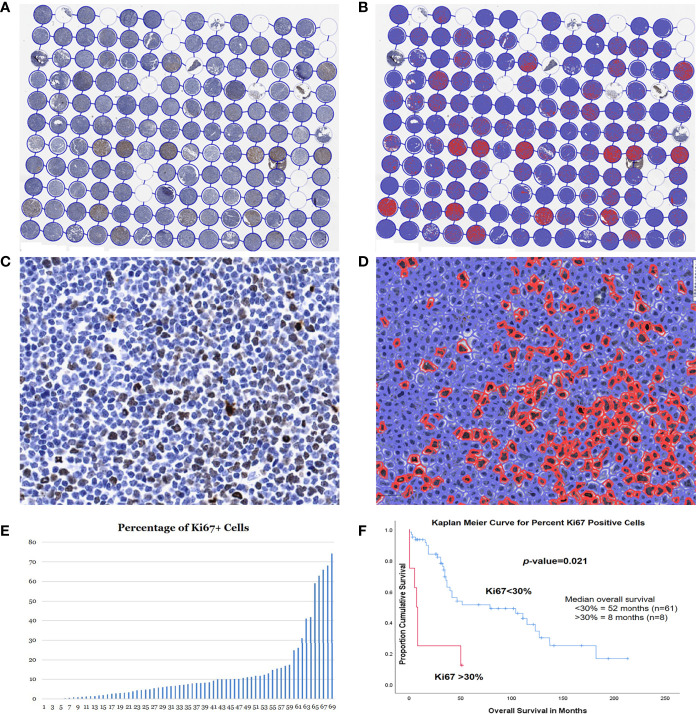
Assessment of Ki67 proliferation rates using QuPath DIA. Whole TMA image with Ki67 immunostain before **(A)** and after **(B)** cell detection and positive cell detection. High magnification of a case before **(C)** and after **(D)** cell detection and positive cell detection. **(E)** Dynamic percentage range of Ki67 proliferation rates of the 73 MCL cases. **(F)** Kaplan-Meier curves for optimal cut-point for patients with high (>30%) *vs*. low (<30%) Ki67 proliferation rates.

A total of 69 MCLs had adequate tissue on the Ki67 stained TMA slide. These 69 patients included 52 males and 17 females, with a median age of 68 years (range 37-92). The median survival was 35 months (range 1-213). The TMA slide stained with Ki67 was scanned using the Aperio scanner and then analyzed using the QuPath program to count the positive cells (**Figures 5A–D**). For each case, the Ki67 proliferation index was assessed using the Ki67-positive cells over all cells in the cores, and an average percentage was calculated from the two cores (**Figure 5E**).

The optimal cut-off point for the Ki67 proliferation index was 31.9% using X-Tile, which was very close to the 30% cut-off in the clinical practice. Therefore, we adopted the same cutoff of 30% for comparison of OS. Most of the cases (61/69, 88%) had a Ki67 proliferation rate of <30%, while the remain 8 cases (12%) >30%. In our study, the patients with lower Ki67 proliferation index of <30% had a median survival of 52 months, which was significantly better than those >30% (median survival 8 months; *p*=0.021) (**Figure 5F**).

The MCL cases with high Ki67 (>30%) had a significantly higher count of CD68+ M1 LAMs than the cases with low Ki67 (<30%) (*p*=0.018). CD163+ M2 LAMs were also increased in the high Ki67 cases than in the low Ki67 group albeit it was not statistically significant (*p*=0.17).

## Discussion

The studies on lymphoma microenvironment (LME) have been markedly increased in recently years, which provided better understanding of the interactions between the neoplastic cells and the supporting cells. In particular, immunotherapies to modulate the signals between the tumor cells and the microenvironmental components have shown promising results in treating lymphomas. This study utilized quantitative imaging analysis on TMA sections to evaluate LAMs and Ki67 proliferation index in MCL. Our findings demonstrated that an increase in CD68+ M1 LAMs or CD163+ M2 LAMs was associated with inferior prognosis in MCL. In addition, M1 LAMs were present in higher numbers than M2 LAMs, indicating that MCL had a lower rate of M1 to M2 polarization in contrast to DLBCL. Both M1 and M2 LAMs were increased in the MCL cases with high Ki67 (>30%), compared to the Ki67 low group (<30%). Furthermore, the M2 LAM counts had a broader range and more frequent low counts, suggesting increased heterogeneity of the M2 microenvironment.

Using multiplexed quantitative immunofluorescence assays, we found that high expression of PD-L1 on M1 LAMs was associated with a slightly improved OS, whereas increased PD-L1 expression on M2 LAMs predicted a slightly inferior OS, although both did not reach statistical significance. Finally, our studies also showed that QuPath DIA is a very promising tool to measure Ki67 proliferation index in MCL, and it accurately separated the patient groups with significantly different OS, which is very close to the 30% cut-off in the clinical practice. Further studies are necessary with large cohorts to validate this assay.

LME consists of variable numbers of immune cells (reactive B-cells, T-cells, macrophages, natural killer cells, and granulocytes), stromal cells, blood vessels, and extracellular matrix (19, 21, 21, 25, 26). The LME influences the behavior of lymphoma, providing a protective niche for neoplastic cells and facilitating tumor cell proliferation and survival. Meanwhile, lymphoma cells recruit and activate the LME cells. The collaborative interactions between lymphoma cells and LME cells enable and sustain tumor cell growth, anti-apoptosis, immunosuppression, angiogenesis, chemoresistance, cell homing and metastasis, and disease progression.

Exploration of the LME has escalated in recent years, particularly with regard to CHL and DLBCL. Similar to other B-cell lymphomas, extrinsic signaling is believed to favor MCL growth, survival, and migration. CD3+, CD8+, and particularly CD4+ T-cells are increased in indolent MCL but decrease with more aggressive histology. A high CD4:CD8 ratio correlates independently from other high-risk prognostic factors with longer OS, suggesting a prognostic role for T-cells in MCL (27). However, studies on the LME cells, soluble factors, intercellular interactions, and intracellular regulations in MCL are limited (18). Therefore, further studies are necessary to integrate the key roles of the LME cells, uncover the mechanisms of the interactions between lymphoma cells and LME, provide more effective treatment, and predict response to therapy and overall survival.

Variable numbers of macrophages are present in nearly all lymphoma types, including CHL, NLPHL, T-cell lymphoma, and low-grade or high-grade B-cell non-Hodgkin lymphoma. These macrophages can be sparsely distributed, form small loose clusters, or become so abundant as to form granulomas and even obscure lymphoma cells. They are referred to as lymphoma associated macrophages (LAMs), which are composed of different biologic subtypes. Furthermore, they can shift their functional phenotypes depending on signals generated from lymphoma and stromal cells, a process known as polarization of LAMs (18, 22, 28). Currently, there are two major subtypes of LAMs, CD68+ M1 and CD163+ M2 LAMs (18, 20, 22, 29–31). M1 LAMs are considered to be tumor-suppressing or classically activated macrophages, and they are mainly involved in inflammatory responses and antitumoral defense by producing various activated lytic enzymes, reactive oxygen species, and inflammation-promoting chemokines. M2 LAMs are known as tumor-promoting, polarized, or alternatively activated macrophages. M2 LAMs secrete key chemokines, cytokines, and bioactive proteases, which can stimulate lymphoma cell growth, angiogenesis, metastasis, chemoresistance, and immunosuppression. In particular, M2 LAMs express checkpoint molecules, including PD1 and PD-L1, which are key immunotherapeutic targets for specific checkpoint-blocking immunotherapies (anti-PD-1/PDL-1). The tumor-promoting M2 LAMs can be induced under the influence of the cytokines (IL-4, IL-13, IL-10 and M-CSF) produced by the lymphoma cells and the microenvironment. Lymphomas may be able to escape the immune surveillance by recruiting and polarizing M1 LAMs to M2 LAMs that highly express immune checkpoint molecules, such as PD-L1 and PD-L2 (20, 22, 30–32).

The presence of LAMs in different lymphoma types may be associated with different outcomes. A high level of macrophages in CHL correlated with EBV-positivity, advanced stage, and inferior prognosis (33). However, in primary testicular DLBCL, high PD-L1+ LAM content predicted favorable survival (34). Primary cutaneous DLBCL, leg type, and nodal DLBCL had a significantly higher level of M2 LAMs than M1 LAMs, which contributed to the poor prognosis (35, 36). According to the studies from Poles et al., EBV-positive DLBCL showed a significant elevated M2 polarization with a higher CD163/CD68 ratio (median value 1.24), compared to EBV-negative DLBCL cohort (median value 0.14) (37). Furthermore, in EBV-negative DLBCL, the CD163/CD68 ratio was higher among advanced-staged/high-tumor burden disease (37). In our study, the overall ratio of CD163:CD68 is 1:3, indicating a lower M2 polarization, in contrast to DLBCL. In addition, the MCL cases with high Ki67 proliferation rates (>30%) contained increased M1 and M2 LAMs, compared to the Ki67 low group (<30%).

One of the most widely studied pathways for immunotherapy is the PD-l and its ligands PD-L1 and PD-L2, which play a critical role in a variety of autoimmune and neoplastic conditions (8–11). PD-1 is expressed on the activated T-cells and B-cells, follicular helper T-cells, dendritic cells, and monocytes/macrophages, while PD-L1 is detected on monocytes/macrophages, dendritic cells, and regulatory T-cells. Many solid tumors (carcinoma and melanoma) and Hodgkin lymphomas express PD-L1. In contrast, PD-L1 is only rarely expressed by non-Hodgkin lymphomas, except some DLBCLs and virus-associated lymphomas. The interaction between PD-1 and PD-L1 reduces T-cell proliferation and cytokine release, inhibits survival proteins, and therefore results in apoptosis. Immune checkpoint inhibitors, such as anti-PD-1 antibody, bind to the PD-1 on activated cytotoxic T-cells, thus stimulating their proliferative capacity and enabling the immune system to resume recognizing, attacking, and destroying tumor cells. This may be one reason that PD-1 inhibition in DLBCL has been effective when directed at specific subtypes, including primary mediastinal large B-cell lymphoma, T-cell/histiocyte-rich large B-cell lymphoma, and EBV-positive lymphoma (15, 16, 36, 38–41). Especially, PD-1 blockade with nivolumab in relapsed and/or refractory CHL has revealed robust response rates as high as 87% (42).

Through multiplex quantitative immunofluorescence analysis, we demonstrated that higher expression of PD-L1 in CD163+ M2 LAMs had a slightly worse OS, whereas higher expression of PD-L1 in CD68+ M1 LAMs was associated with a slightly better OS, although both did not reach statistical significance. These findings suggested that the tumor-suppressing functions in M1 LAMs and tumor-promoting functions in M2 LAMs are at least partially attributed to the expression of PD-L1 in these two subtypes. Studies have shown that PD-L1 expressed on MCL was able to inhibit T-cell proliferation induced by the tumor cells, impair the generation of antigen-specific T-cell responses, and render MCL cells resistant to T-cell-mediated cytolysis (17). In addition, blocking or knocking down PD-L1 on MCL cells enhanced T-cell responses and restored tumor-cell sensitivity to T-cell-mediated killing *in vitro* and *in vivo* (17). Moreover, knocking down PD-L1 on MCL cells primed more CD4+ or CD8+ memory effector T cells. Therefore, it might be beneficial to include checkpoint-blocking immunotherapies for aggressive, relapsed or chemo-resistant MCLs, particularly in those cases with high PD-L1 expression on neoplastic cells and M2 LAMs.

In a recently published study using syngeneic MCL cells and xenografted human MCL cell lines in the mouse models, Le et al. confirmed the presence of polarized M1 and M2 TAMs in the MCL tumors *in vivo*. They also demonstrated that MCL cells can differentiate TAM toward a M2-like phenotype and particularly M2 but not M1 TAMs favor MCL cell growth and tumorigenesis *via* STAT1 signaling by secretion of IL-10 (43). In another study, through coculture of MCL cells and monocytes, Papin et al. showed that MCL polarized monocytes into M2-like macrophages through secretion of CSF1 and, to a lesser extent, IL-10, which in turn promoted lymphoma survival and proliferation (44). These studies explored molecular level of the dynamic interactions between MCL cells and TAMs in the lymphoma microenvironment.

In our study, we also assessed the Ki67 proliferation rates of MCL by an automated method using the QuPath DIA. The Ki67 immunostaining is commonly utilized to assess the proliferation index of MCL (cut-off at 30%). The Ki67 proliferation index is a prognostic biomarker independent of sMIPI score and predicts survival in patients receiving chemotherapy and autologous stem cell transplant; a low Ki67 correlates with a more indolent form of MCL. However, it may be difficult to accurately assess the Ki67 proliferation rate, since the current “eyeballing” method has a high inter-observer variability and often results in over-estimation.

Automated immunohistochemical scoring by computerized image analysis (CIA) enables accurate and reproducible scores by circumventing the poor reproducibility of manual scoring. The automated scoring system also provides extensive evaluation of relevant cutoff points, taking advantage of the continuous scale quantification compared to the categorical measurement of manual scoring. In our study, the QuPath DIA accurately separated the patient groups with significantly different OS, and the optimal cut-off point for the Ki67 proliferation index was 31.9%, which was very close to the 30% cut-off in the clinical practice. Therefore, QuPath DIA is a very promising tool to measure Ki67 proliferation index in MCL. Further studies may be necessary with large cohorts to validate this assay, which may potentially be applied for future practice.

Taken together, we utilized the complexed quantitative fluorescence imaging analysis with automated whole-slide imaging and integrated whole-slide image analysis in our studies. These techniques enabled simultaneous detection and automatic quantification of multiple markers on TMA sections constructed from formalin-fixed paraffin-embedded tissues. However, our studies are limited by the small number of MCL cases in the different cohorts and the heterogeneity of treatments.

## Conclusions

M1 and M2 LAM counts may serve as a fast and affordable tool to stratify MCL patients into risk groups. MCL had a significantly lower rate of M1 to M2 polarization, and the high levels of M1 and M2 LAMs were associated with poor OS. However, high expression of PD-L1 on M1 and M2 LAMs may have different impacts on MCL outcomes, which requires further studies with larger cohorts and more in-depth assessment of LAMs.

## Author’s Note

The content of this manuscript has been presented in part as abstracts at the United States and Canadian Academy of Pathology (USCAP) Annual Meetings, 02/28/2020-03/05/2020, Los Angeles, CA. (Abstracts #1319 and #1415) [Abstracts from USCAP 2020: Hematopathology. Mod Pathol 33, 1409–1586 (2020)].

## Data Availability Statement

The original contributions presented in the study are included in the article/**Supplementary Material**. Further inquiries can be directed to the corresponding author.

## Author Contributions

ZP designed the study, collected and analyzed the data, and wrote the paper. PL collected and analyzed the data, performed the assays, and revised the manuscript. JY designed the study, collected and analyzed the data, and revised the manuscript. FA performed the assays and analyzed the data. AM analyzed the data and revised the manuscript. KF, GY, and HC collected the data. MX designed the study and revised the manuscript. DR designed the study. All authors contributed to the article and approved the submitted version.

## Conflict of Interest

MX has served in the Seattle Genetics lymphoma advisory board and as a Pure Marrow consultant. DR has served as a consultant, advisor or served on a Scientific Advisory Board for Amgen, Astra Zeneca, Agendia, Biocept, BMS, Cell Signaling Technology, Cepheid, Daiichi Sankyo, GSK, Merck, NanoString, Perkin Elmer, PAIGE, and Ultivue. He has received research funding from Astra Zeneca, Cepheid, Nanostring, Navigate/Novartis, NextCure, Lilly, Ultivue, and Perkin Elmer.

The remaining authors declare that the research was conducted in the absence of any commercial or financial relationships that could be construed as a potential conflict of interest.

## Publisher’s Note

All claims expressed in this article are solely those of the authors and do not necessarily represent those of their affiliated organizations, or those of the publisher, the editors and the reviewers. Any product that may be evaluated in this article, or claim that may be made by its manufacturer, is not guaranteed or endorsed by the publisher.
